# The *ADCYAP1R1* Gene Is Correlated With Posttraumatic Stress Disorder Symptoms Through Diverse Epistases in a Traumatized Chinese Population

**DOI:** 10.3389/fpsyt.2021.665599

**Published:** 2021-06-07

**Authors:** Li Wang, Jingyi Zhang, Gen Li, Chengqi Cao, Ruojiao Fang, Ping Liu, Shu Luo, Guangyi Zhao, Yingqian Zhang, Kunlin Zhang

**Affiliations:** ^1^Laboratory for Traumatic Stress Studies and Center for Genetics and BioMedical Informatics Research, CAS Key Laboratory of Mental Health, Institute of Psychology, Chinese Academy of Sciences, Beijing, China; ^2^Department of Psychology, University of Chinese Academy of Sciences, Beijing, China; ^3^People's Hospital of Deyang City, Deyang, China

**Keywords:** *ADCYAP1R1*, posttraumatic stress disorder, HPA axis, SNP, gene-gene interaction

## Abstract

The adenylate cyclase activating polypeptide 1 (pituitary) receptor (*ADCYAP1R1*) gene is associated with the hypothalamic-pituitary-adrenal (HPA) axis, which controls stress responses. The single-nucleotide polymorphism of *ADCYAP1R1*, rs2267735, has been investigated in many studies to test its association with posttraumatic stress disorder (PTSD), but the results have not been consistent. It is worth systematically exploring the role of rs2267735 in PTSD development. In this study, we analyzed rs2267735 in 1,132 trauma-exposed Chinese individuals (772 females and 360 males). We utilized the PTSD checklist for DSM-5 (PCL-5) to measure the PTSD symptoms. Then, we analyzed the main, G × E (rs2267735 × trauma exposure), and G × G (with other HPA axis gene polymorphisms) effects of rs2267735 on PTSD severity (total symptoms). There were no significant main or G × E effects (*P* > 0.05). The G × G *ADCYAP1R1*-*FKBP5* interaction (rs2267735 × rs1360780) was associated with PTSD severity (beta = −1.31 and *P* = 0.049) based on all subjects, and the G × G *ADCYAP1R1*-*CRHR1* interaction (rs2267735 × rs242924) was correlated with PTSD severity in men (beta = −4.72 and *P* = 0.023). Our study indicated that the *ADCYAP1R1* polymorphism rs2267735 may affect PTSD development through diverse gene-gene interactions.

## Introduction

### *ADCYAP1R1*, an HPA Axis Gene, Is Associated With Stress Response

The pituitary adenylate cyclase-activating polypeptide (PACAP) and its receptor (PAC1) are widely distributed in hypothalamic and limbic structures (1–6). This peptide and receptor help regulate responses to anxiety-provoking stimuli or stress (3, 7–10). The *ADCYAP1R1* gene, which encodes the receptor PAC1, is associated with the neuroendocrine system hypothalamic–pituitary–adrenal (HPA) axis that is involved in controlling mammalian stress responses (11, 12).

There are many single-nucleotide polymorphisms (SNPs) in *ADCYAP1R1*. One of them, rs2267735, was reported to play a role in anxiety symptoms, fear-related cognitions, and stress-related responses (13–15). For example, rs2267735 could regulate the normal stress response by affecting the bind of estrogen receptors alpha and estrogen response element (16).

### PTSD Is a Stress-Related Mental Disorder Related to *ADCYAP1R1* and Other HPA Axis Genes

Posttraumatic stress disorder (PTSD) is a trauma- and stressor-related disorder (17) that can occur after exposure to traumatic events (18). Many studies have shown that PTSD is associated with *ADCYAP1R1* and *ADCYAP1R1*–environment interactions [e.g., (19–22)]. In a Chinese population, a significant association between *ADCYAP1R1* and the PTSD emotional numbing cluster was found, a finding that extended previous results (23).

Moreover, PTSD is correlated with other genes that are associated with the HPA axis or regulation of its activity. The *FKBP5* gene regulates the HPA axis by encoding FK506 binding protein 5 (FKBP5). *FKBP5* can bind to glucocorticoid receptors (24). The *FKBP5* gene was detected to have a significant correlation with PTSD in a meta-analysis study (25, 26) and could reflect the risk of co-morbidity of PTSD and depression in mild trauma exposure (27). *FKBP5*-environment interactions predicted the risk of PTSD in adults (28–30). The *CRHR1* gene and the *CRHR2* gene regulate the HPA axis together by binding their encoding products, corticotropin-releasing hormone (CRH) receptors CRF1 and CRF2, to CRH. The *CRHR1* gene has been suggested to be associated with PTSD (31–36). The *CRHR2* gene has been shown to significantly affect PTSD (32, 37, 38) as well as through a gene–environment interaction (39).

### Associations Between *ADCYAP1R1* and PTSD Are Worth Further Exploration, and Genetic Studies Should Consider Main G × E and G × G Effects

Numerous neurogenetic studies of the *ADCYAP1R1* gene have been performed. Some of them gave out a significant main effect on PTSD. For example, Ressler and her colleagues have found that *ADCYAP1R1* could contribute to the diagnosis of PTSD and the severity of PTSD symptoms in females who suffered a major trauma (21). Later, Lind et al. (20) used a meta-analysis to show that the C allele at rs2267735 of *ADCYAP1R1* significantly increased the risk of PTSD in women. A review supported as well that the epigenetic regulation of *ADCYAP1R1* might predict PTSD risk (40). However, not all studies performed the same, and they even led to no effects. In order to replicate the finding of Ressler et al. (21), two independent samples were newly selected, and their results failed to repeat such (41). In addition, a recent investigation did not show that *ADCYAP1R1* was strongly correlated with PTSD (42). The possible reasons for why these study results have not been consistent may be due to the types of trauma exposure (43, 44) and the sample characteristics (23, 41). However, the underlying factors have been most likely not singular. Therefore, we need more studies to enrich the evidence of the main or gene-environment effects of *ADCYAP1R1* on PTSD.

Furthermore, the four genes *ADCYAP1R1, FKBP5, CRHR1*, and *CRHR2* are all associated with PTSD and have a close relationship with each other by regulating the HPA axis together. Since genes functionally interact with each other, a phenomenon that is usually called epistasis (i.e., gene-gene interaction) in genetics (45), the gene-gene interactions between *ADCYAP1R1* and other HPA axis genes need to be investigated. However, to our knowledge, a neurogenetic study that investigates the gene–gene interactions between *ADCYAP1R1* and other HPA axis genes in PTSD has not been conducted. Thus, the correlation between the interactions of *ADCYAP1R1* with other HPA axis genes (including *FKBP5, CRHR1*, and*CRHR2*) and PTSD is worth further exploring.

### Brief Study Design

To further explore the role of *ADCYAP1R1* in PTSD development and the physiological underpinnings of the stress response, this study examined the main, G × E (rs267735 × trauma exposure), and G × G (with other HPA axis gene polymorphisms) effects of rs2267735 on PTSD severity in a cohort of 2008 Chinese Wenchuan earthquake survivors.

## Materials and Methods

### Participants

In the present study, we selected 1,132 survivors of the Wenchuan earthquake in Hanwang Town, Sichuan Province, China. These participants have been described previously (38, 46), and we provide the detailed information for the cohort here again for convenience.

The participants were adults (older than 18 years old) without any mental illness or intellectual disability history. Regarding self-reported gender results, 68.19% of the participants were women, and the remaining participants were men. Moreover, most participants reported that their ethnicities were Han (99.65%), and the remaining were reported to be Qiang. Furthermore, the marital status of 13.07% of the participants was unmarried. In addition, 32.50% of the participants had an educational level involving high school or above.

The measurement occurred in a large rebuilt community of Hanwang Town from November 6–8 in 2013. The investigators were all trained clinical psychologists, psychotherapists, and psychology graduate students. Our study was approved by the institutional review board of the Institute of Psychology, Chinese Academy of Sciences, and was completely in compliance with national legislation and the Declaration of Helsinki. All the participants signed informed consent forms.

### Measures

Earthquake-related trauma exposure was assessed by a self-reported questionnaire (46). The participants needed to answer 10 yes (1) or no (0) questions about whether they experienced (a) being trapped under a rubble, (b) being injured, (c) being disabled due to injuries, (d) participating in rescue efforts, (e) witnessing the death of someone, (f) being exposed to mutilated bodies, (g) the traumatic death of a family member, (h) the traumatic injury of a family member, (i) the traumatic death of a friend or neighbor, and (j) losing livelihood due to a disaster. The level of trauma exposure was defined as the sum score of the 10 items.

PTSD symptoms were assessed by a self-reported questionnaire, the PTSD Checklist of DSM-5 [PCL-5; (47, 48)]. The 20 items included on the PCL-5 used a five-point Likert scale (from 0 = never to 4 = most of the time). The Chinese version has shown good validity and reliability (49). The participants needed to complete the PCL-5 Chinese version according to their PTSD symptom occurrence frequency and severity in the past month of the earthquake. The final score indicating PTSD symptom severity was the sum of all the item scores.

Depressive symptoms were measured by a self-report questionnaire, the Center for Epidemiological Studies-Depression (CES-D) scale (50). The 20 items included on the CES-D used a four-point Likert scale (from 0 = rare or none of the time/ <1 day to 3 = most or all of the time/5–7 days). The Chinese version has been validated and widely used in Chinese populations (51). The participants were required to answer the Chinese version of the questionnaire on the basis of their personal experiences in the past week. The final score indicating depressive symptoms was reflected by the sum of all the item scores.

### SNP Selection and Genotyping

Four HPA axis genes with nine relevant SNPs were selected for study: *ADCYAP1R1* (rs2267735), *FKBP5* (rs9296158, rs3800373, rs1360780, and rs9470080), *CRHR1* (rs4458044 and rs242924), and *CRHR2* (rs8192496 and rs2267715). These SNPs were genotyped in a previous study (38); thus, we directly used their data.

We used PLINK (52) to perform the Hardy–Weinberg equilibrium (HWE) test (53). In addition, we also calculated the SNP call rate and minor allele frequency (MAF).

### Statistical Analyses

PLINK 1.07 and R 3.4.4 (https://www.r-project.org/) were used to conduct all the statistical analyses in our study. A pairwise comparison of demographic variables and the SNP rs2267735 was tested by analysis of variance (ANOVA) in R. A linkage disequilibrium (LD) analysis has been done previously (38), and we directly referenced those results.

The associations between rs2267735 (coded as minor allele counts per subject) and PTSD severity were detected by a linear regression model and adjusted for age, sex (1: women and 0: men), education level (1: high school or above and 0: other), marital status (1: single and 0: married), the environmental factor trauma exposure, and depressive symptoms. The main, gene–environment interaction (G × E), and gene–gene interaction (G × G) effects were considered. When studying the G × E effects, we further added a linear model analysis with all gene–covariate and environment–covariate interactions (54). The all-interactions model was used to control for potential confounders and compared to the single-interaction model. A subset analysis for women and men was performed to detect potential sex differences. All *P*-values of linear regressions in our study are two-sided.

For adjustment of possible bias and correction for multiple comparisons, the SNP-related variables were subjected to a permutation test with 100,000 cycles. The results with significant *P* and permutation *P-*values are reported. Moreover, we calculated the effect size (semipartial correlation) as well.

## Results

### Overall Results

The summary of trauma exposure, PTSD severity (total symptom) scores, and depressive symptoms (CES-D scale total score) are shown in Table 1. The mean value of depressive symptoms in all samples is 37.00, and the standard error of the mean was 8.63. According to the DSM-5, we inferred probable PTSD in our participants. As our likely diagnoses showed, the probable PTSD prevalence was 13.78% (14.89% for women and 11.38% for men). All of the above-mentioned data were exactly the same as those of our previous study (38) since they were based on the same cohort, and we provided the data here for convenience.

**Table 1:** Summary of posttraumatic stress disorder (PTSD) severity (total symptoms), trauma exposure, and depressive symptom scores.

	**Minimum**	**1st quartile**	**Median**	**3rd quartile**	**Maximum**	**Mean ± SD**
PTSD severity	1	9	16	26	77	18.76 ± 13.46
Trauma exposure	0	2	3	5	10	3.47 ± 1.84
Depressive symptoms	20	31	36	42	68	37.00 ± 8.63

The HWE test indicated that the genotype frequencies of rs2267735 agreed with the Hardy–Weinberger equilibrium (*P* = 0.812). The call rate was 100%, and the MAF (for allele G) was 0.485. The pairwise comparisons of the demographic variables and SNP rs2267735 are shown in Supplementary Table 1. There was no significant result between the SNP and depression.

Based on previous LD analysis results (38), during the following analyses, we used rs2267735 × rs1360780 to index the G × G effect of *ADCYAP1R1*–*FKBP5* and rs2267735 × rs242924 to index the G × G effect of *ADCYAP1R1*–*CRHR1*.

### Main Effects and G × E Effects of *ADCYAP1R1* on PTSD Severity

As shown, the minor allele G of rs2267735 of *ADCYAP1R1* was not significantly associated with PTSD severity. The G × E *ADCYAP1R1*–environment (rs2267735 × trauma exposure) effect on PTSD severity was also not significant. The trauma exposure was measured by a 10-item scale in the present study [see Supplementary Tables 2, 3 (single-interaction model) and Supplementary Table 4 (all-interaction model) for details].

### G × G Effects of *ADCYAP1R1* Genes on PTSD Severity

**Figure 1:**
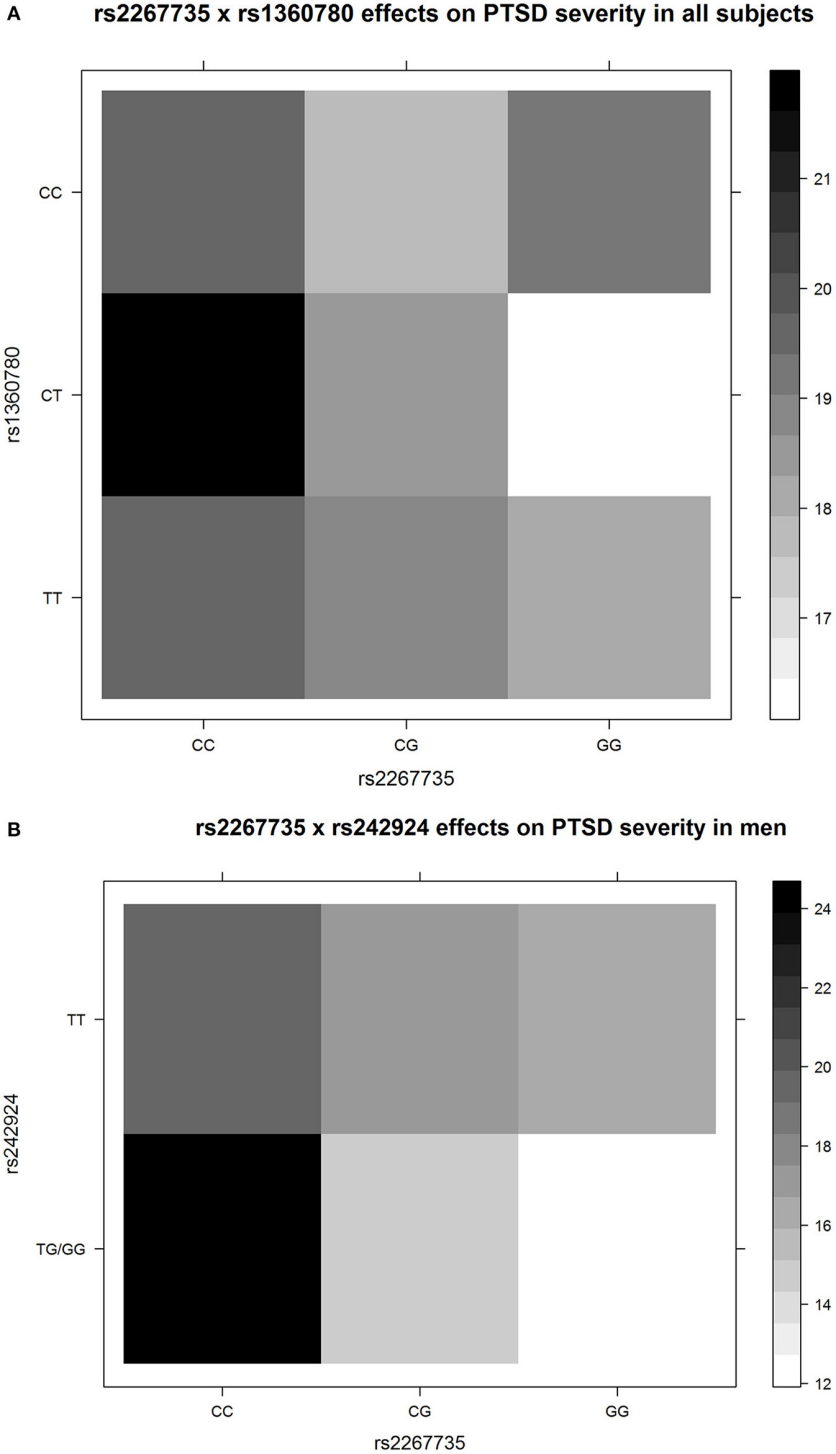
The G × G (epistasis) effects represented by the distributions of posttraumatic stress disorder (PTSD) severity across genotype combinations. The colors of each unit were filled according to the means of PTSD severity of the subjects with the corresponding specific genotype combinations. The upper panel **(A)** shows rs2267735 × rs1360780 for all subjects, and the lower panel **(B)** shows rs2267735 × rs242924 for men. Only three men had the rs242924 genotype GG; thus, we merged GG and TG. The figures directly show epistasis. For example, in **(A)**, for the subjects with rs1360780 genotype CC, the minor allele G of rs2267735 is not correlated to PTSD severity; for the subjects with rs1360780 genotypes CT and TT, the minor allele G of rs2267735 corresponds to decreased PTSD severity, with different effect sizes. The figures were drawn by R3.4.4.

The G × G *ADCYAP1R1*–*FKBP5* (rs2267735 × rs1360780) effect on PTSD severity was significant in all samples (*P* = 0.049). The G × G *ADCYAP1R1*–*CRHR1* (rs2267735 × rs242924) effect on PTSD severity was significant in men (*P* = 0.023; see Tables 2, 3 for details). Figure 1 shows the G × G (epistasis) effects represented by the distributions of PTSD severity across genotype combinations. We also provide all the other results of the G × G analysis (Supplementary Table 5).

**Table 2:** G × G effects of *ADCYAP1R1*–*FKBP5* (rs2267735 × rs9296158, rs3800373, rs1360780, and rs9470080) on posttraumatic stress disorder severity.

**Single-nucleotide polymorphism**	**Sample**	**Beta (95% CI)**	**Standard error**	***t*-value**	***P*-value**	***P*_perm_**	**Cochran's *P***	***I*^**2**^**	**Effect size (semipartial correlation)**
rs9296158	All	−0.901 (−2.171, 0.369)	0.648	−1.389	0.165	0.16423	0.674	0	−0.032
	Females	−1.121 (−2.679, 0.437)	0.795	−1.410	0.159	0.15752			−0.040
	Males	−0.541 (−2.748, 1.666)	1.126	−0.481	0.631	0.62810			−0.020
rs3800373	All	−1.215 (−2.524, 0.0943)	0.668	−1.818	0.069	0.06881	0.517	0	−0.042
	Females	−1.554 (−3.155, 0.0473)	0.817	−1.902	0.058	0.05642			−0.053
	Males	−0.630 (−2.923, 1.663)	1.170	−0.539	0.590	0.58629			−0.022
rs1360780	All	−1.316 (−2.628, −0.005)	0.669	−1.969	0.049	0.04909	0.668	0	−0.045
	Females	−1.556 (−3.159, 0.048)	0.817	−1.904	0.057	0.05645			−0.053
	Males	−0.937 (−3.240, 1.365)	1.171	−0.801	0.421	0.42086			−0.033
rs9470080	All	−0.923 (−2.205, 0.359)	0.654	−1.410	0.159	0.15875	0.996	0	−0.033
	Females	−0.969 (−2.545, 0.607)	0.804	−1.204	0.229	0.2278			−0.034
	Males	−0.962 (−3.177, 1.253)	1.130	−0.851	0.395	0.39455			−0.035

*P_perm_, permutation P-value*.

**Table 3:** G × G effects of *ADCYAP1R1–CRHR1* (rs2267735 × rs4458044 and rs242924) on posttraumatic stress disorder severity.

**Single-nucleotide polymorphism**	**Sample**	**Beta (95% CI)**	**Standard error**	***t*-value**	***P*-value**	***P*_perm_**	**Cochran's *P***	***I*^**2**^**	**Effect size (semipartial correlation)**
rs4458044	All	1.183 (−0.209, 2.5746)	0.710	1.667	0.096	0.09314	0.529	0	0.039
	Females	0.722 (−1.0498, 2.494)	0.904	0.799	0.425	0.42008			0.022
	Males	1.637 (−0.595, 3.869)	1.139	1.437	0.152	0.14898			0.059
rs242924	All	−1.232 (−3.662, 1.197)	1.238	−0.995	0.317	0.31568	0.027	79.4	−0.023
	Females	1.016 (−2.052, 4.085)	1.563	0.650	0.516	0.51643			0.018
	Males	−4.725 (−8.828, −0.621)	2.086	−2.265	0.023	0.02461			−0.092

*P_perm_, permutation P-value*.

## Discussion

### Summary of Results

In this study, two gene–gene interactions (G × G) were identified. An *ADCYAP1R1*–*FKBP5* effect was associated with PTSD severity in all subjects, and an *ADCYAP1R1*–*CRHR1* effect was found in men.

An *FKBP5*-environment interaction and *ADCYAP1R1* have been found to be associated with PTSD (19–22, 28–30), but to our knowledge, few studies have shown a G × G effect of *FKBP5* and *ADCYAP1R1*. Therefore, this is the first study showing that the *ADCYAP1R1* polymorphism rs2267735 could affect PTSD development through a novel *ADCYAP1R1*–*FKBP5* interaction. Moreover, this finding was observed under the conditions of earthquake trauma type and in a Chinese ethnic group.

In addition, *CRHR1* has been reported in prospective studies to moderate childhood maltreatment effects on PTSD symptoms (28, 30, 31). The gene–gene–environment (*CRHR1* × *5–HTTLPR* × childhood maltreatment) interaction could predict adult depressive symptoms among black people of lower socioeconomic status (55). Here the identified G × G effect between *ADCYAP1R1* and *CRHR1* on PTSD in men was interesting and further revealed the influence of gene–gene interactions on PTSD.

### An Indication of the Results of G × G Effects From a Physiological Perspective

Earlier findings of PTSD candidate gene studies have shown that rs2267735 in *ADCYAP1R1* could increase amygdala reactivity and reduce functional connectivity between the amygdala and hippocampus (21, 56). In addition, four SNPs in *FKBP5* (rs9296158, rs3800373, rs1360780, and rs9470080), which are related to regulating the stress response system, were shown to increase amygdala reactivity to threat stimuli and the severity of PTSD symptoms in adulthood (28, 57). Thus, the *ADCYAP1R1*–*FKBP5* interaction may influence PTSD development by affecting amygdala reactivity together.

Both *ADCYAP1R1* and *CRHR1* are involved in stress response by modulating CRF function and the release of cortisol through the adrenal cortex (58). Therefore, the *ADCYAP1R1*–*CRHR1* interaction suggests that their gene expression may influence PTSD by regulating CRF together.

### One G × G Was Sex Nonspecific, and the Other One Was Related to Sex

The genotypes of rs2267735 were differentially distributed in females and males (Supplementary Table 2). To explore sex differences in the two G × G (*ADCYAP1R1*–*FKBP5* and *ADCYAP1R1*–*CRHR1*) interactions, we used Cochran's *Q* statistic test by PLINK. To investigate heterogeneity between results from women and men (i.e., the sex–G × G interactions), we also calculated the heterogeneity index *I*^2^ (range, 0–100). The *ADCYAP1R1*-*FKBP5* interactions were not different between women and men (Cochran's *Q* statistic *P* < 0.668 and *I*^2^ = 0), which indicated that the effects were sex non-specific. The *ADCYAP1R1*-*CRHR1* interactions were different between sexes (Cochran's *Q* statistic *P* < 0.027 and *I*^2^ = 79.4), which showed that sex might moderate the effects. Many studies have shown that sex could regulate the relationship between *ADCYAP1R1* and PTSD. Women with the C allele at rs2267735 of *ADCYAP1R1* had a higher PTSD risk than those with the G allele (20, 21). Moreover, a genome-wide association study indicated that *CRHR1* is a genetic factor related to PTSD reexperiencing symptom (33). Our findings suggested that *ADCYAP1R1* might also affect PTSD in men in a specific manner by interacting with *CRHR1*.

### The Limitations of This Study

Our study had some limitations. One was the scales used in the current study. The PTSD was measured by a self-reported scale, and a clinical assessment could be used in the future. The trauma exposure was measured by a 10-item scale. Although it has shown great reliability and validity (38, 46, 59–61), other questionnaires with more items could be tried to be applied further. Depression was regarded as a covariate, so it was mentioned less in the study. The second was that the sample size was relatively small, and the number of women was approximately two times that of men. Large sample sizes and sex-balanced samples should be considered. The single ethnic group and trauma exposure type also need to be broadened. The third was about the definition of the G × E effect. Because this was a candidate gene study, so the “G” was only considered as specific genotype instead of genetics, and the “E” was only concentrated on earthquake, which was the most related environment factor to the PTSD symptom measured in the present study. Other possible related factors, such as rearing environment, social support, socioeconomic status, parenting styles, and education, could be taken into consideration in the future. The fourth was the sex difference of *ADCYAP1R1*–*CRHR1* interactions which might be due to the sex hormone level which was different between men and women. This point could be detected through physiological experiments and indications.

## Conclusions

We have provided additional genetic findings regarding the HPA axis and its involvement in PTSD and indicated that the *ADCYAP1R1* polymorphism rs2267735 may affect PTSD development through diverse gene–gene interactions. Our analysis provided new insight into PTSD genetics.

## Data Availability Statement

The original contributions presented in the study are included in the article/Supplementary Material, further inquiries can be directed to the corresponding author/s.

## Ethics Statement

The studies involving human participants were reviewed and approved by the institutional review board of the Institute of Psychology, Chinese Academy of Sciences. The patients/participants provided their written informed consent to participate in this study.

## Author Contributions

LW and KZ conceived and designed the overall study. LW collected the samples. KZ, JZ, YZ, and GZ performed the statistical analysis. CC maintained genotyping. PL and SL contributed to collecting samples. KZ, JZ, and LW wrote the manuscript. GL and RF helped revise the manuscript. All the authors read and approved the final version of the manuscript.

## Conflict of Interest

The authors declare that the research was conducted in the absence of any commercial or financial relationships that could be construed as a potential conflict of interest.
