# Supplementary material for: The ADCYAP1R1 Gene Is Correlated With Posttraumatic Stress Disorder Symptoms Through Diverse Epistases in a Traumatized Chinese Population
Source: Front Psychiatry. 2021 Jun 7;12:665599. doi: 10.3389/fpsyt.2021.665599 (PMC8216487; doi:10.3389/fpsyt.2021.665599)
Supplement: Supplementary file 1 [file Data_Sheet_1.PDF]

Supplementary Table 1 Results of pairwise comparisons between demographic variables and rs2267735 (by ANOVA or χ^2 test).

Demography SNP	Sex	Age	Education	Marital	Depress	Trauma Total
rs2267735	χ^2 = 8.149, *df* =2, *P* = 0.017*	$F_{(2,1129)}$ = 0.1115, *P* = 0.891	χ^2 = 3.017, *df* =2, *P* = 0.221	χ^2 = 1.198, *df* =2, *P* = 0.549	$F_{(2,1129)}$ = 1.540, *P* = 0.215	$F_{(2,1129)}$ = 0.625, *P* = 0.535

Supplementary Table 2 Main effects of *ADCYAP1R1* (rs2267735) on PTSD severity.

Sample	Beta (95% CI)	Std. Error	*t* value	*P* value	P_{perm}	Cochran's P	I^2	Effect size (semipartial correlation)
All	-0.748 (-1.62, 0.12)	0.444	-1.684	0.092	0.09289	0.213	35.4	-0.039
Females	-0.282 (-1.36, 0.80)	0.551	-0.511	0.609	0.61223			-0.014
Males	-1.438 (-2.90, 0.03)	0.748	-1.923	0.055	0.0557			-0.078

†P_{perm}, permutation *P* value.

Supplementary Table 3 The G×E effects of *ADCYAP1R1* (rs2267735×trauma exposure) on PTSD severity (single interaction model).

Sample	Beta (95% CI)	Std. Error	*t* value	*P* value	P_{perm}	Cochran's P	I^2	Effect size (semipartial correlation)
All	-0.152 (-0.63, 0.32)	0.243	-0.623	0.533	0.56148	0.752	0	-0.014
Females	-0.048 (-0.65, 0.56)	0.308	-0.157	0.875	0.87949			-0.004
Males	-0.208 (-0.99, 0.58)	0.401	-0.519	0.604	0.65328			-0.018

†P_{perm}, permutation *P* value.

Supplementary Table 4 The G×E effects of *ADCYAP1R1* (rs2267735×trauma exposure) on PTSD severity (all-interaction model).

Sample	Beta (95% CI)	Std. Error	*t* value	*P* value	P_{perm}	Cochran's P	I^2	Effect size (semipartial correlation)
All	-0.119 (-0.62, 0.38)	0.254	-0.468	0.640	0.64971	0.757	0	-0.011
Females	-0.032 (-0.65, 0.59)	0.315	-0.101	0.919	0.92138			-0.003
Males	-0.200 (-1.07, 0.67)	0.443	-0.452	0.652	0.68978			-0.018

†P_{perm}, permutation *P* value.

Supplementary Table 5 G×G effects of *ADCYAP1R1 – CRHR2*(rs2267735 × rs8192496 and rs2267715) on PTSD severity.

SNP	Sample	Beta (95% CI)	Std. Error	*t* value	*P* value	P_{perm}	Cochran's *P*	I^2	Effect size (semipartial correlation)
rs8192496	All	0.715 (-0.494, 1.924)	0.617	1.159	0.247	0.24732	0.872	0	0.027
	Females	0.666 (-0.845, 2.177)	0.771	0.864	0.388	0.38661			0.024
	Males	0.458 (-1.567, 2.483)	1.033	0.443	0.658	0.6618			0.018
rs2267715	All	-0.002 (-1.219, 1.215)	0.621	-0.003	0.998	0.99671	0.856	0	0.000
	Females	-0.168 (-1.654, 1.318)	0.758	-0.222	0.825	0.82912			-0.006
	Males	0.071 (-2.044, 2.186)	1.079	0.065	0.948	0.95672			0.003

†P_{perm}, permutation *P* value.
